# Changes to women’s childbirth plans during the COVID-19 pandemic and posttraumatic stress symptoms: a cross-national study

**DOI:** 10.1007/s00737-023-01403-3

**Published:** 2023-12-16

**Authors:** Ana Mesquita, Raquel Costa, Pelin Dikmen-Yildiz, Susana Faria, Gabriela Silvestrini, Vera Mateus, Eleni Vousoura, Claire A. Wilson, Ethel Felice, Erilda Ajaz, Eleni Hadjigeorgiou, Camellia Hancheva, Yolanda Contreras-García, Sara Domínguez-Salas, Emma Motrico, Isabel Soares, Susan Ayers

**Affiliations:** 1 School of Psychology, CIPsi, University of Minho, Braga, Portugal; 2 ProChild CoLab Against Poverty and Social Exclusion – Association (ProChild CoLAB) Campus de Azurém, 4800-058, Guimarães, Guimarães, Portugal; 3 EPIUnit – Instituto de Saúde Pública, Universidade do Porto, Rua das Taipas, n° 135, 4050-600 Porto, Portugal; 4 Laboratório para a Investigação Integrativa e Translacional em Saúde Populacional (ITR), Universidade do Porto, Rua das Taipas, n° 135, 4050-600 Porto, Portugal; 5 Hei-Lab: Digital Human-Environment Interaction Lab, Lusófona University, Porto, Portugal; 6 Department of Psychology, Kirklareli University, Kirklareli, Turkey; 7 Centre of Mathematics, Department of Mathematics, University of Minho, Guimarães, Portugal; 8 Center for Research in Neuropsychology and Cognitive Behavioral Intervention, Faculty of Psychology and Educational Sciences, University of Coimbra, Coimbra, Portugal; 9 Department of Psychology, School of Philosophy, National and Kapodistrian University of Athens, Athens, Greece; 10 Institute of Psychiatry, Psychology and Neuroscience, King’s College London and South London and Maudsley NHS Foundation Trust, London, UK; 11 Department of Psychiatry, University of Malta, Msida, Malta; 12 Department of Education and English Language, Beder University College, Tirana, Albania; 13 Department of Nursing, School of Health Science, Cyprus University of Technology, Limassol, Cyprus; 14 Sofia University “St. Kliment Ohridski”, Sofia, Bulgaria; 15 Departamento de Obstetricia y Puericultura Facultad de Medicina, Universidad de Concepción, Concepción, Chile; 16 Departamento de Psicología Experimental, Universidad de Sevilla, Sevilla, Spain; 17 Department of Psychology, University of Loyola, Sevilla, Spain; 18 Centre for Maternal and Child Health Research School of Health and Psychological Sciences, City, University of London, London, United Kingdom

**Keywords:** Birth, Childbirth plan, Postpartum, Posttraumatic stress disorder, Mental health

## Abstract

A considerable number of women giving birth during COVID-19 pandemic reported being concerned about changes to their childbirth plans and experiences due to imposed restrictions. Research prior to the pandemic suggests that women may be more at risk of post-traumatic stress symptoms (PTSS) due to unmet expectations of their childbirth plans. Therefore, this study aimed to examine if the mismatch between women’s planned birth and actual birth experiences during COVID-19 was associated with women’s postpartum PTSS. Women in the postpartum period (up to 6 months after birth) across 11 countries reported on childbirth experiences, mental health, COVID-19-related factors, and PTSS (PTSD checklist DSM-5 version) using self-report questionnaires (ClinicalTrials.gov: NCT04595123). More than half (64%) of the 3532 postpartum women included in the analysis reported changes to their childbirth plans. All changes were significantly associated with PTSS scores. Participants with one and two changes to their childbirth plans had a 12% and 38% increase, respectively, in PTSS scores compared to those with no changes (Exp(β) = 1.12; 95% CI [1.06–1.19]; *p* < 0.001 and Exp(β) = 1.38; 95% CI [1.29–1.48]; *p* < 0.001). In addition, the effect of having one change in the childbirth plan on PTSS scores was stronger in primigravida than in multigravida (Exp(β) = 0.86; 95% CI [0.77–0.97]; *p* = 0.014). Changes to women’s childbirth plans during the COVID-19 pandemic were common and associated with women’s postpartum PTSS score. Developing health policies that protect women from the negative consequences of unexpected or unintended birth experiences is important for perinatal mental health.

**Supplementary information:**

The online version contains supplementary material available at 10.1007/s00737-023-01403-3.

## Introduction

Giving birth is usually associated with joy and happiness but can be a challenging and sometimes even traumatic experience for a significant number of women (Leinweber et al. 2022). The coronavirus disease 2019 (COVID-19) pandemic compromised this unique event even further by bringing about unprecedented changes in daily lives and creating major disruption in healthcare services (Kotlar et al. 2021). The highly contagious and potentially fatal nature of COVID-19 required sudden and unexpected changes in daily habits, including mandatory use of face masks, lockdowns, travel, and transport restrictions that caused heightened levels of uncertainty, stress, and fear (Salari et al. 2020). Reduction in the quality of maternal and newborn care (MNC) around the time of childbirth due to the COVID-19 pandemic was reported by a considerable number of women giving birth even in healthcare facilities in high-income countries (Lazzerini et al. 2022). Unsurprisingly, health care professionals, health care services users and their carers, friends, and families were more affected by the psychological distress associated with the pandemic when more restrictive COVID-19 mitigation measures (e.g., use of personal protective equipment, frequent COVID-19 testing, visitor restrictions) were implemented and the risk for transmission was greatest (Thomas and Suresh 2022).

In order to prevent or control the spread of COVID-19, healthcare facilities introduced specific policies and restrictions to services, including in MNC services. A considerable number of women giving birth during the COVID-19 pandemic were concerned about or reported changes to their childbirth plans or birth settings specifically due to the pandemic (Aydin et al. 2022; Gildner and Thayer 2020). Those changes included having limited or no access to birth facilities (e.g., birthing ball or water birth (Hui et al. 2020; Vazquez-Vazquez et al. 2021)) or pain relief (Hui et al. 2020; Liu et al. 2021), shortened period from admission to giving birth until discharge (Gildner and Thayer 2020; Vazquez-Vazquez et al. 2021), not being able to have their initially planned mode of birth (Liu et al. 2021), and increased demand for community birth or giving birth at a different hospital than initially planned (Gildner and Thayer 2020; Grünebaum et al. 2022; Vazquez-Vazquez et al. 2021). Other examples of changes reported by women were directly related to pandemic-specific measures, e.g., restricted presence of a birth partner or companion during labor and birth, suspension or exclusion of visitors after birth (Aydin et al. 2022; Gildner and Thayer 2020; Lazzerini et al. 2022), mandatory face mask wearing during labor and delivery (Shuman et al. 2022), having COVID-19 testing before their scheduled admission time (Shuman et al. 2022), and limited availability or unavailability and/or limited support from health care providers (HCPs) (Horsch et al. 2020; Lazzerini et al. 2022; Liu et al. 2021).

During pregnancy, women develop plans and expectations related to the mode of birth, childbirth setting, pain intensity and relief, and support from significant others or HCPs (Staneva 2013). Women’s expectations of childbirth are strongly associated with childbirth experiences (Soet et al. 2003; Verreault et al. 2012). How and whether childbirth expectations match with the actual childbirth experience may play a role on how women experience childbirth (Pirdil and Pirdel 2016). Research has shown that unfulfilled women’s expectations in non-pandemic scenarios are associated with lower childbirth satisfaction and increased PTSS; however, the association with depression and fear of birth is inconsistent (Webb et al. 2021). The authors emphasized the need for further research to understand how women’s childbirth plans are met, and its importance for childbirth experiences and psychological outcomes (Webb et al. 2021). Research on this topic is of particular relevance during the COVID-19 pandemic, which, due to the unpredictable nature of childbirth and uncertainty around childbirth events, may have led to increased levels of birth trauma and PTSS.

A study conducted in the USA with women who gave birth during the COVID-19 pandemic reported that 72.3% were partially symptomatic (endorsing at least one of the diagnostic criteria A–E) and 5.9% of the women fulfilled the diagnostic criteria for childbirth-related posttraumatic stress disorder (PTSD) (Diamond and Colaianni 2022). A recent systematic review of 154 studies reported rates of childbirth-related PTSD of 4.7% and PTSS of 12.3% (Heyne et al. 2022), whereas specifically during the COVID-19 pandemic, the reported rates of clinically significant postpartum PTSS ranged between 12.5 and 29.4% (Liu et al. 2021; Ostacoli et al. 2020). This is unsurprising, as several COVID-19-related factors have been associated with PTSD symptoms, including mother–baby separation before hospital discharge, restrictions on significant others’ support, mandatory face mask use during labor and childbirth, and changes to birth setting and breastfeeding plans (Diamond and Colaianni 2022; Liu et al. 2021). Notably, the risk for PTSS/PTSD and its consequences might be substantially increased in high-risk women, e.g., women with suspected/confirmed COVID-19 (Mayopoulos et al. 2020). This is a concern because of the potential negative impact of postpartum PTSD on adaptation to motherhood, couple relationship, and mother–infant bonding (McKenzie-McHarg et al. 2015).

In the context of the COVID-19 pandemic, it is possible to speculate that the gap between women’s childbirth plans and their actual childbirth experiences was widened due to the MNC services restrictions, and to the numerous and rapidly changing healthcare policies and uneven implementation of those policies (Lalor et al. 2021). Therefore, this study aims to examine if changes to women’s childbirth plans—conceptualized as changes to expected care and experiences—during the COVID-19 pandemic were associated with women’s postpartum PTSS. Specific objectives were to analyze (1) the cumulative effect of changes in childbirth plans on PTSS and (2) the potential moderators of the effect of changes in childbirth plans on PTSS.

## Methods

### Study design

This cross-sectional study was part of an international prospective observational cohort study in which pregnant and postpartum women from 14 countries (Albania, Argentina, Brazil, Bulgaria, Chile, Cyprus, France, Greece, Israel, Malta, Portugal, Spain, Turkey, and the UK) were invited to complete an online survey about their perinatal experiences and feelings during the COVID-19 pandemic. The project was disseminated through social media, organizational networks (universities, health centers, NGOs working with perinatal mental health), policymakers, stakeholders, and networks of colleagues and acquaintances of the research team.

### Participants

Women with a child up to 6 months old, aged 18 years old or older, living in one of the 11 of the 14 countries of the consortium were eligible for this analysis (Argentina, France, and Israel were not included due to lack of data in the variables of interest).

Between June 7 and October 31 of 2020, a total of 5895 women in the postpartum period completed the questionnaire, gave their informed consent, and were considered eligible for participating in the study. From those, 1772 failed to complete the PTSD checklist DSM-5 version or the questions regarding changes in childbirth experiences due to the COVID-19 pandemic, and 591 did not report the date of childbirth or reported a date before March 2020, and therefore were not included in the analysis, yielding a final sample of 3532 women.

### Measures

#### Sociodemographic and clinical characteristics

The online survey included an adapted version (Motrico et al. 2021) of the Coronavirus Perinatal Experiences – Impact Survey (COPE-IS) (Thomason et al. 2020). As a newly developed measure, psychometric properties of COPE-IS are yet to be established. The COPE questionnaires were originally available in English and translated to Spanish and German. Researchers from each country included in the study performed the translation and cultural adaptation of the questionnaires from English into the official language of their country, following several methodological steps, defined a priori, and detailed in Motrico et al. (2020).

The demographic information included age, country of birth, educational level (secondary education or lower/higher education), professional status (employed/unemployed), and first pregnancy (yes/no). Health-related data included exposure to COVID-19-related problems (yes/no), history of mood and/or anxiety disorders (yes/no), and current treatment for mental health problems (yes/no).

#### Changes to women’s childbirth plans

In order to measure the changes to childbirth plans—here conceptualized as changes to women’s expected care and experiences—information of the COPE-IS was extracted from the question “Did any of your childbirth plans change as a result of the COVID-19 outbreak?”, in which women could select multiple items: (1) reduced access to preferred medications before or after delivery (i.e., nitrous oxide, epidural); (2) change to planned delivery location; (3) my elective induction or C-section was not allowed as planned; (4) my elective vaginal birth changed to induction or C-section; (5) my HCP (e.g., doctor, doula, midwife) was not available for my baby’s birth as planned; (6) support people (e.g., partner, family) were not allowed to attend baby’s delivery; (7) I was separated from the baby immediately after delivery; (8) I was separated from the baby for a long period after delivery (e.g., my baby was quarantined in the hospital nursery; not skin to skin contact); (9) No changes.

#### Posttraumatic stress symptoms

The survey included a subset of 10 questions from the PTSD checklist DSM-5 version (PCL-5), adapted to the COVID-19 pandemic. The original PCL-5 has 20 items and refers to PTSD symptoms in the last 7 days using a Likert rating scale (0 = Not at all to 4 = Extremely) (Bovin et al. 2016). In this study, we used the following 10 items: (1) Feeling super alert or watchful or on guard; (2) Feeling jumpy or easily startled; (3) Having difficulty concentrating; (4) Trouble experiencing positive feelings; (5) Feeling guilty or blaming yourself; (6) Feeling irritable, angry, or aggressive; (7) Repeated disturbing and unwanted thoughts about the COVID-19 outbreak; (8) Repeated disturbing dreams about the COVID-19 outbreak; (9) Trying to avoid information or reminders about the COVID-19 outbreak; (10) Taking too many risks or doing things that could cause you harm. This subset was used to avoid redundancy with other items in the survey and represents each DSM-5 criterion for PTSD covered in the original PCL-5 (Kinser et al. 2021). Answers were reported in a 5-point Likert scale (0 = Not at all to 4 = Extremely). The PTSS score is a sum of the 10 items with total scores ranging from 0 to 40, as a one-factor solution is adequate (Motrico et al. 2023). Higher scores indicate greater symptom severity. Reliability in this sample was good (Cronbach’s alpha = 0.883, 95% CI = [0.877–0.889]).

### Statistical analysis

Survey data were manually checked for accuracy and consistency. Descriptive analysis was performed for the dataset as a whole and by country, including socio-demographics, mental health–related information, and PTSS score. Continuous variables were expressed as medians and interquartile range (IQR), and categorical variables as their absolute frequency and percentage.

To examine the association between changes to childbirth plans and experiences and the PTSS score, Tweedie Compound Poisson Generalized Linear Mixed Models were estimated. These models have been applied in a wide range of fields in which continuous data with exact zeros regularly arise (Zhang 2013). As observations collected from the same country are often correlated, countries were treated as random effects.

In addition, GLMMs were conducted to test whether exposure to COVID-19-related problems, current mental health concerns, history of mood and/or anxiety disorder, unemployment, gravidity (number of pregnancies: primigravida vs. multigravida), maternal education, and age moderated the association between the number of changes to childbirth plans and PTSS.

Statistical analyses were performed using R programming language (Core Team 2021). We used glmmTMB package to perform generalized linear mixed-effects modeling. *P* values lower than 0.05 were considered statistically significant.

## Results

Participant’s characteristics are shown in Table 1. The average age was 32 (4.9 SD) years; 59.5% were primigravida, 14.5% were unemployed, and 26.0% had secondary or lower educational level. In addition, 16.6% had a history of mood and/or anxiety disorders and 6.1% were currently having treatment for mental health problems. Ten percent of participants had already experienced COVID-19 related symptoms.

**Table 1:** Sociodemographic and clinical characteristics of participants (*N* = 3532)

		Postpartum women		
		Primigravida *N* = 2103	Multigravida *N* = 1429	Total *N* = 3532
PTSS score Median (Q1–Q3)		8 (4–15)	8 (4–14)	8 (4–15)
Maternal age *N* (%)	≤ 24	173 (9.3)	48 (3.7)	221 (7.0)
	25–35	1356 (73.2)	772 (60.0)	2128 (67.8)
	≥ 36	323 (17.4)	467 (36.3)	790 (25.2)
	*Missing*	*251 (11.9)*	*142 (9.9)*	*393 (11.1)*
Mean (SD) Median (Q1–Q3)		31 (4.8) 31 (28–34)	34 (4.7) 34 (31–37)	32 (4.9) 32 (29–36)
Currently on treatment for mental health problems *N* (%)	Yes	119 (5.9)	89 (6.4)	208 (6.1)
	No	1899 (93.4)	1283 (92.5)	3182 (93.0)
	Declined to answer	15 (0.7)	15 (1.1)	30 (0.9)
	*Missing*	*70 (3.3)*	*42 (2.9)*	*112 (3.1)*
Exposure to COVID-19-related problems *N* (%)	Yes	163 (7.8)	190 (13.3)	353 (10.0)
	No *Missing*	1940 (92.2) *0 (0.0)*	1239 (86.7) *0 (0.0)*	3179 (90.0) *0 (0.0)*
History of mood and/or anxiety disorder *N* (%)	Yes	370 (17.6)	215 (15.0)	585 (16.6)
	*No*	1733 (82.4)	1214 (85.0)	2947 (83.4)
	*Missing*	*0 (0.0)*	*0 (0.0)*	*0 (0.0)*
Mother education *N* (%)	Secondary education or lower	457 (21.7)	379 (26.5)	836 (26.0)
	Higher education	1445 (68.7)	931 (65.1)	2376 (74.0)
	*Missing*	*201 (9.6)*	*119 (8.4)*	*320 (9.0)*
Unemployment *N* (%)	Yes	313 (14.9)	198 (13.9)	511 (14.5)
	No	1790 (85.1)	1231 (86.1)	3021 (85.5)
	*Missing*	*0 (0.0)*	*0 (0.0)*	*0 (0.0)*
Country *N* (%)	Albania	9 (0.4)	4 (0.3)	13 (0.4)
	Brazil	317 (15.1)	260 (18.2)	577 (16.3)
	Bulgaria	23 (1.1)	11 (0.8)	34 (1.0)
	Chile	141 (6.7)	141 (9.9)	282 (8.0)
	Cyprus	138 (6.6)	86 (6.0)	224 (6.3)
	Greece	233 (11.1)	97 (6.8)	330 (9.3)
	Malta	77 (3.7)	40 (2.8)	117 (3.3)
	Portugal	391 (18.6)	269 (18.8)	660 (18.7)
	Spain	267 (12.7)	217 (15.2)	484 (13.7)
	Turkey	357 (17.0)	174 (12.2)	531 (15.0)
	UK	150 (7.1)	130 (9.1)	280 (7.9)
	*Missing*	*0 (0.0)*	*0 (0.0)*	*0 (0.0)*

The overall PTSS scores median was 8 (interquartile range [4–15]; Supplementary Table 1). Women from Brazil and Chile had the highest median (12 [7–20] and 10 [5–18], respectively), while women from Albania and Turkey had the lowest median (6 [1–9] and 6 [3–12], respectively). Sixty-four percent reported at least one change to the childbirth plan. The most frequently reported change to childbirth plans was “Support people (e.g., partner, family) were not allowed to attend baby’s delivery” (41.5%). The least frequently reported change was “My elective induction or C-section was not allowed as planned” (2.0%). Frequency of changes to childbirth plans are detailed in Table 2.

**Table 2:** Change on childbirth plans overall and by gravidity (*N* = 3532)

	Primigravida *N* = 2103	Multigravida *N* = 1429	Total *N* = 3532
	*N* (%)	*N* (%)	*N* (%)
1. Reduced access to preferred medications before or after delivery (i.e., nitrous oxide, epidural)	82 (3.9)	47 (3.3)	129 (3.7)
2. Change to planned delivery location	255 (12.1)	169 (11.8)	424 (12.0)
3. My elective induction or C-section was not permitted as planned	40 (1.9)	30 (2.1)	70 (2.0)
4. My elective vaginal birth changed to induction or C-section	265 (12.6)	112 (7.8)	377 (10.7)
5. My health care provider (e.g., doctor, doula, midwife) was not available for my baby’s birth as planned	162 (7.7)	102 (7.1)	263 (7.4)
6. Support people (e.g., partner, family) were not permitted to attend baby’s delivery	965 (45.9)	500 (35.0)	1465 (41.5)
7. I was separated from baby immediately after delivery	223 (10.6)	107 (7.5)	330 (9.3)
8. I was separated from baby for a long period after delivery (e.g., my baby was quarantined in the hospital nursery; not skin to skin contact)	96 (4.6)	40 (2.8)	136 (3.9)
9. No change	682 (32.4)	578 (40.4)	1260 (35.7)
10. Other	381 (18.1)	251 (17.6)	632 (17.9)

Adjusted models, addressing the individual role of each change on childbirth plans (while controlling to all the other changes), showed that all changes to childbirth plans were significantly associated with PTSS scores except “Change to planned delivery location” (Exp(β) = 1.05, 95% CI [0.98–1.13]; *p* = 0.223) and “I was separated from the baby for a long period after delivery (e.g., my baby was quarantined in the hospital nursery; no skin to skin contact)” (Exp(β) = 1.08, 95% CI [0.94–1.24]; *p* = 0.262; Table 3). The association with PTSS scores was strongest for those reporting “Reduced access to preferred medications before or after delivery (i.e., nitrous oxide, epidural)” (Exp(β) = 1.23, 95% CI [1.08–1.40]; *p* = 0.002) and “My elective induction or C-section was not allowed as planned” (Exp(β) = 1.23, 95% CI [1.05–1.45]; *p* = 0.013).

**Table 3:** Fixed effects estimates from generalized linear mixed model for PTSS score associated with changes in childbirth plans due to COVID-19 (***N*** = 3532)

	Unadjusted		Adjusted*	
	PTSS score		PTSS score	
CHANGES IN CHILDBIRTH PLANS	Exp(β) (95% CI)	*p* value	Exp(β) (95% CI)	*p* value
Reduced access to preferred medications before or after delivery (i.e., nitrous oxide, epidural)	1.34 (1.18–1.53)	< 0.001	1.23 (1.08–1.40)	0.002
Change to planned delivery location	1.12 (1.03–1.20)	0.005	1.05 (0.98–1.13)	0.223
My elective induction or C-section was not permitted as planned	1.40 (1.19–1.65)	< 0.001	1.23 (1.05–1.45)	0.013
My elective vaginal birth changed to induction or C-section	1.26 (1.16–1.36)	< 0.001	1.17 (1.08–1.27)	< 0.001
My health care provider (e.g., doctor, doula, midwife) was not available for by baby’s birth as planned	1.24 (1.13–1.35)	< 0.001	1.14 (1.09–1.24)	0.006
Support people (e.g., partner, family) were not be permitted to attend baby’s delivery	1.21 (1.15–1.28)	< 0.001	1.15 (1.09–1.22)	< 0.001
I was separated from baby immediately after delivery	1.26 (1.16–1.36)	< 0.001	1.10 (1.00–1.21)	0.034
I was separated from baby for a long period after delivery (e.g., my baby was quarantined in the hospital nursery; not skin to skin contact)	1.25 (1.10–1.42)	< 0.001	1.08 (0.94–1.24)	0.262

^*^Adjusted for all the other variables assessing changes in childbirth plans

### Cumulative effects of changes to childbirth plan on PTSS scores

The number of women reporting one change in their childbirth plans was 1233 (34.9%; 771 primigravida) and two or more changes were reported by 771 women (21.8%; 508 primigravida). Participants who experienced two or more changes in their childbirth plans had PTSS scores 38% higher than those that had no changes (Exp(β) = 1.38; 95% CI [1.29–1.48]; *p* < 0.001). Participants with one change to their childbirth plans had PTSS scores about 12% higher than those that had no changes (Exp(β) = 1.12; 95% CI [1.06–1.19]; *p* < 0.001).

### Moderation effects of changes on childbirth plan on PTSS

The association between changes in childbirth plans and PTSS scores was moderated by gravidity (see Table 4). The effect of having one change in the childbirth plan on PTSS scores was stronger in primigravida than in multigravida (Exp(β) = 0.86; 95% CI [0.77–0.97]; *p* = 0.014). However, the effect of having two or more changes in the childbirth plan on PTSS scores was similar for both primigravida and multigravida (Exp(β) = 0.88; 95% CI [0.77–1.00]; *p* = 0.067).

**Table 4:** Fixed effects estimates from generalized linear mixed model for PTSS score, testing as moderators gravidity, COVID-19 symptoms, previous history of mental health problems, current treatment to mental health problems, unemployment, and education and country as a random effect

Fixed effects	Exp(β) (95% CI)	*p* value
Gravidity (*N* = 3532)		
Intercept	8.29 (7.40–9.30)	< 0.001
Childbirth plan (ref: no change)		
Childbirth plan (one change)	1.19 (1.10–1.29)	< 0.001
Childbirth plan (two or more changes)	1.45 (1.34–1.58)	< 0.001
Gravidity (ref: primigravida)		
Gravidity (multigravida)	1.07 (0.99–1.16)	0.091
Childbirth plan (one change) × gravidity (multigravida)	0.86 (0.77–0.97)	0.014
Childbirth plan (two or more changes) × gravidity (multigravida)	0.88 (0.77–1.00)	0.067
COVID-19 symptoms (*N* = 3532)		
Intercept	9.70 (8.28–11.36)	< 0.001
Childbirth plan (ref: no change)		
Childbirth plan (one change)	1.18 (0.99–1.40)	0.067
Childbirth plan (two or more changes)	1.59 (1.33–1.91)	< 0.001
COVID-19 symptoms (ref: no)		
COVID-19 symptoms (yes)	0.87 (0.76–0.99)	0.039
Childbirth plan (one change) × COVID-19 symptoms (yes)	0.94 (0.78–1.13)	0.507
Childbirth plan (two or more changes) × COVID-19 symptoms (yes)	0.84 (0.69–1.01)	0.066
History of mental health problems (*N* = 3532)		
Intercept	7.91 (7.18–8.72)	< 0.001
Childbirth plan (ref: no change)		
Childbirth plan (one change)	1.12 (1.05–1.19)	< 0.001
Childbirth plan (two or more changes)	1.36 (1.27–1.47)	< 0.001
History of mental health problems (ref: no)		
History of mental health problems (yes)	1.54 (1.40–1.69)	< 0.001
Childbirth plan (one change) × history of mental health problems (yes)	1.04 ( 0.90–1.20)	0.585
Childbirth plan (two or more changes) × history of mental health problems (yes)	0.97 (0.84–1.12)	0.707
Current treatment for mental health concerns (*N* = 3420)		
Intercept	11.71 (9.92–13.85)	< 0.001
Childbirth plan (ref: no change)		
Childbirth plan (one change)	1.64 (0.94–1.45)	0.170
Childbirth plan (two or more changes)	1.46 (1.17–1.80)	< 0.001
Current treatment for mental health concerns (ref: no)		
Current treatment for mental health concerns (yes)	0.72 (0.62–0.83)	< 0.001
Childbirth plan (one change) × current treatment for mental health concerns (yes)	0.95 (0.76–1.19)	0.678
Childbirth plan (two or more changes) × current treatment for mental health concerns (yes)	0.93 ( 0.74–1.16)	0.519
Unemployment (*N* = 3532)		
Intercept	8.37 (7.49–9.35)	< 0.001
Childbirth plan (ref: no change)		
Childbirth plan (one change)	1.14 (1.07–1.22)	< 0.001
Childbirth plan (two or more changes)	1.40 (1.30–1.50)	< 0.001
Unemployment (ref: no)		
Unemployment (yes)	1.20 (1.06–1.35)	0.003
Childbirth plan (one change) × unemployment (yes)	0.86 (0.73–1.02)	0.089
Childbirth plan (two or more changes) × unemployment (yes)	0.90 (0.76–1.07)	0.240
Level of education (*N* = 3212)		
Intercept	10.06 (8.84–11.45)	< 0.001
Childbirth plan (ref: no change)		
Childbirth plan (one change)	1.00 (0.89–1.13)	0.894
Childbirth plan (two or more changes)	1.31 (1.16–1.49)	< 0.001
Level of education (ref: secondary or lower)		
Level of education (higher)	0.83 (0.76–0.92)	< 0.001
Childbirth plan (one change) × level of education (higher)	1.12 (0.97–1.28)	0.115
Childbirth plan (two or more changes) × level of education (higher)	1.03 (0.89–1.19)	0.681

A history of mental health problems, current treatment for mental health problems, unemployment, exposure to COVID-19-related problems, and education did not moderate the association between changes in the childbirth plans and PTSS scores (see Table 4).

## Discussion

### Main findings

This study examined whether changes to women’s childbirth plans during the COVID-19 pandemic were associated with postpartum PTSS. Results confirmed that changes to childbirth plans were common during the COVID-19 pandemic, and more than half of the women (64%) experienced at least one change to their childbirth plans. This had a significant impact on women, with significant increases in PTSS reported by those who experienced changes. There was a dose–response effect of changes in childbirth plans on postpartum PTSS, with PTSS scores increasing from 12% in women exposed to one change to 38% in women with cumulative exposure to two or more changes. This dose–response effect of the exposure to changes in childbirth plans is consistent with the notion of the allostatic load (i.e., cumulative impact of exposure to stress) being associated with greater PTSD scores and other negative health outcomes (Carbone et al. 2022). Primigravida seemed to be more susceptible to lower exposures (i.e., exposure to one change) compared to multigravida, yet for exposure to multiple changes (i.e., two or more) there was no difference in PTSS scores.

The most common change to childbirth plans was related to the unexpected ban of having a support person attending the delivery, reported by 41.5% of the women. A smaller number of women (7.4%) reported not having their planned HCP with them at the birth. These changes conflict with extensive evidence showing the importance of having the support of a significant person during labor and childbirth on physical and psychological outcomes for women and their infants. For example, it is well established that continuous support during birth leads to improved maternal and infant outcomes (Bohren et al. 2017). Reviews of clinical trials show that continuous support during labor is associated with less pain medication, shorter labors, fewer cesarean births, and greater satisfaction with birth (Bohren et al. 2017). Conversely, poor support or interpersonal difficulties during birth are a key risk factor for postpartum PTSS/PTSD (Ayers et al. 2016; Harris and Ayers 2012). PTSS has been associated with social support–related factors, such as poor interaction with HCPs, perceptions of inadequate care during birth, low support from partner and staff, and being poorly informed or not listened to (Creedy et al. 2000; Czarnocka and Slade 2000; Soet et al. 2003). These unexpected events, including the lack of support either from caregivers and/or partners in such an important moment of women’s life, may have increased the feelings of insecurity and uncertainty or fear for their own health or their newborns’ health, and have been described as a strong predictor of PP-PTSS (Ayers et al. 2016). In addition, in the postnatal period there are dynamic structural and functional changes that take place in women’s brain that are not exclusively adaptive, and increase the vulnerability for the development of mental disorders (Barba-Müller et al. 2019), which is particularly relevant in such unpredictable pandemic context. It is therefore concerning that so many women were not able to have their partner or other support person at the time of childbirth during COVID-19, as also reported in other studies (Lazzerini et al. 2022).

The greatest increased in PTSS scores was for women who planned to have an elective induction or C-section but were not allowed to. MNC services and/or HCPs not permitting an induction or C-section was relatively rare (reported by 2% of women) but was associated with a 40% increase in PTSS scores. Similarly, women who planned to have a vaginal birth but had an induction or C-section had a 26% increase in PTSS scores. In addition, women who were unable to have their preferred pain relief also had one of the largest increased PTSS scores. This highlights the importance of complying with the planned mode of birth – not the mode of birth per se, but the one that women desired and planned. This is consistent with previous reports of increased PTSS in women who planned a C-section birth but ending up having a vaginal birth, compared to women who planned and had a vaginal birth (Garthus-Niegel et al. 2014). This greatest PTSS scores in women who planned an induction or C-section is probably due to specific reasons related to individual factors, such as previous traumatic birth experiences, obstetric risk or complications, and/or fear of childbirth. Denying the preferred/planned mode of birth is therefore likely to cause anxiety and distress, increasing the likelihood of a traumatic birth and of developing PTSS.

Unexpected separation from the newborn for a long period after delivery was associated with PTSS scores in the unadjusted analyses. However, once analyses were adjusted for all the other changes in childbirth plans, women who were separated from the newborn for a long period after delivery had a small increase in PTSS scores. We were expecting a greater effect of the separation of the baby since it decreases the skin-to-skin contact and breastfeeding which are associated with increased PTSS (Chen et al. 2022; Kahalon et al. 2022) possibly through the effects of oxytocin, bonding, and memory consolidation (Deforges et al. 2022; Witteveen et al. 2020). It is difficult to interpret our findings since we do not have information on the reasons or characteristics of the separation. More research is therefore needed to better understand these associations and the mechanisms underlying them.

Finally, having a changed location of birth was associated with increased PTSS scores. However, when assessing the impact of changing the location of birth controlling for the effect of other changes to the childbirth plan, the effect was no longer significant.

## Strengths and limitations

A strength of this study is that it reports changes to women’s childbirth plans during the COVID-19 pandemic in a large European sample of women from 11 countries. However, the cross-sectional nature of the survey and the use of an online convenience sampling limit the conclusions regarding causality and the generalizability of the results. Most women in our sample were highly educated and employed. Unfortunately, we did not collect information on ethnicity so are unable to report how representative the sample is in relation to ethnicity. A final limitation is that the measure of PTSS was shortened for this study, so it is not validated and does not allow assessment of diagnostic criteria for PTSD. However, the measure was shortened from a widely used, validated scale (PCL-5).

## Conclusion

This study clearly shows that changes to women’s childbirth plans during the COVID-19 pandemic were associated with greater postpartum PTSS, indicating that restrictions to women’s planned childbirth had a critical negative impact on women’s mental health. In the uncertainty and unpredictability of the COVID-19 pandemic, there were understandable reasons underpinning the restrictions in MNC services. It is therefore important to learn from experiences and research during this time to inform evidence-based post-pandemic MNC in regular and in crisis situations that may arise in the future.

### Supplementary information

Below is the link to the electronic supplementary material.

Supplementary file1 (DOCX 15 KB)

## Data Availability

The data is available upon reasonable request to the authors.
