# Supplementary material for: Changes to women’s childbirth plans during the COVID-19 pandemic and posttraumatic stress symptoms: a cross-national study
Source: Arch Womens Ment Health. 2023 Dec 16;27(3):393–403. doi: 10.1007/s00737-023-01403-3 (PMC11116223; doi:10.1007/s00737-023-01403-3)
Supplement: Supplementary file 1 — Supplementary file1 (DOCX 15 KB) [file 737_2023_1403_MOESM1_ESM.docx]

| **Country** | **PTSS score**  **Median [Q25-Q75]** |
| --- | --- |
| Albania | 6 [1-9] |
| Brazil | 12 [7-20] |
| Bulgaria | 8.5 [4-14.75] |
| Chile | 10 [5-18] |
| Cyprus | 7 [3-13] |
| Greece | 7.5 [3-13] |
| Malta | 7 [3-10] |
| Portugal | 7 [3-13] |
| Spain | 9 [4-15] |
| Turkey | 6 [3-12] |
| United Kingdom | 8 [4-13] |
| Total | 8 [4-15] |

Supplementary Table 1 – Post-traumatic stress symptoms (PTSS) scores overall and by country
